# Analysis of risk factors for pneumonia in patients with catatonia: a cross-sectional analysis

**DOI:** 10.3389/fpsyt.2024.1430194

**Published:** 2024-09-27

**Authors:** Qingpeng Meng, Danna Zhou, Xixi Zhao, Jing Wang, Li Yin, Sixiang Liang, Xiao Ji

**Affiliations:** The National Clinical Research Center for Mental Disorders & Beijing Key Laboratory of Mental Disorders Beijing Anding Hospital & the Advanced Innovation Center for Human Brain Protection, Capital Medical University, School of Mental Health, Beijing, China

**Keywords:** psychiatric emergency department, catatonia, aspiration pneumonia, risk factors, nursing care

## Abstract

**Objective:**

The clinical management of catatonia has always been a focus of psychiatric nursing. Unfortunately, there is still limited research on the risk factors and nursing methods for patients with catatonia and bacterial pneumonia. Few studies have identified and analyzed the clinical risk factors for catatonia patients with bacterial pneumonia. This study aims to explore the risk factors and preventive nursing measures for pneumonia in patients with catatonia.

**Methods:**

A total of 88 patients with catatonia treated in the emergency department of a psychiatric hospital from January 2019 to October 2021 were selected. They were divided into bacterial pneumonia group (n=17) and non-pneumonia group (n=71) based on whether they had pneumonia. The demographic data and clinical characteristics of the two groups were compared. Logistic regression analysis and point-biserial correlation were used to analyze the risk factors for developing pneumonia in patients with catatonia.

**Results:**

The incidence of pneumonia in patients with catatonia was 19.32%. Correlation analysis showed that age (r=0.216, p=0.043), The Activities of Daily Living Scale (ADL) score (r=0.265, p=0.013), cell count of white blood (r=0.591, p<0.001), neutrophil count (r=0.599, p<0.001), percentage of neutrophils (r=0.311, p=0.003), C-reactive protein (r=0.558, p<0.001), bedridden days (r=0.470, p<0.001), and albumin level (r=-0.288, p=0.007) were significantly associated with pneumonia. Multivariate logistic regression analysis showed that smoking, bedridden days, family support, and nutritional status were risk factors for pneumonia in patients with catatonia.

**Conclusion:**

Reducing smoking and bedridden days, improving nutrition, and providing timely preventive nursing care by family members can reduce the occurrence of pneumonia in patients with catatonia.

## 1 Introduction

Catatonia is a psychomotor syndrome characterized by motor, emotional, and behavioral symptoms, which are significantly associated with physical illnesses and mental disorders (1). Catatonia was first described by Kahlbaum as a state of psychomotor inhibition and behavioral abnormalities, manifested as numbness, silence, negativity, and stereotypy (2). Previous reports have shown that catatonia is present in 4%-67% of patients with schizophrenia and 14%-71% of patients with mood disorders (3). The prevalence of catatonia has also been reported to be 7-45%, depending on the treatment environment (4–6). Patients with catatonia often present to psychiatric emergencies due to their stiffness and resistance, but the difficulty in cooperating with both psychiatric and physical examinations makes early diagnosis challenging (7). Under-diagnosed catatonia was more frequent in the presence of agitation, echolalia, or grimacing, and under-recognition of catatonia may relate to physician unawareness of the heterogeneous cluster of signs and symptoms that constitute this syndrome (4). Relevant studies have indicated that up to 59% of patients with catatonia symptoms go unrecognized or are underdiagnosed, with 37% of them not receiving adequate treatment (4), and severe cases may lead to death (5, 8). Despite having normal limb strength, patients with catatonia are unable to move normally (9), with the most common symptoms being resistance, silence, agitation, and stiffness (10).

The clinical management of catatonia has always been a focus of psychiatric nursing, and previous studies have attempted to analyze its comorbidities. Research has shown that patients with catatonia have significantly higher levels of C-reactive protein (CRP) compared to patients with other mental disorders, suggesting a clear association between catatonia and inflammation, not solely due to changes in psychopathology or stress levels (11). Compared to patients with other mental disorders, there is a significantly higher incidence of physical diseases such as bedsores, pressure ulcers, infections, and venous thrombosis (2). It has been reported that respiratory tract infections are the main sites of hospital-acquired infections in patients with mental disorders, accounting for as high as 79.31% (12). Pneumonia is a frequently occurring comorbidity in patients with catatonia. Due to prolonged bed rest, reduced food intake, and weakened immune function, these patients are more prone to pneumonia. The main reason is that the lower part of the lungs is in a state of congestion and edema due to prolonged stasis, leading to difficulty in expectorating respiratory secretions, which accumulate in the small and medium bronchi and serve as an excellent culture medium for bacteria, thus making it highly susceptible to developing consolidative pneumonia (13). The prognosis of pneumonia in general patients is related to factors such as age, nutritional status, comorbidities, severity of pneumonia, and timely and effective treatment (14). Patients with catatonia have a worse prognosis when combined with pneumonia due to poor treatment compliance, impaired self-care abilities (partially or completely dependent on others), poor nutritional status, and compromised immune system function dominated by psychiatric symptoms. These factors may all contribute to worsening of the infection or poor prognosis, posing a threat to the patient’s life. At the same time, prolonged treatment of pneumonia-related physical diseases can exacerbate the condition of patients with catatonia, greatly influencing the judgment and treatment of catatonia symptoms. Therefore, nursing care for patients with catatonia accompanied by pneumonia is of utmost importance.

Unfortunately, there is still limited research on the risk factors and nursing methods for patients with catatonia and pneumonia. Few studies have identified and analyzed the clinical risk factors for catatonia patients with pneumonia. Identifying these risk factors can help clinical professionals recognize the risk of pneumonia in patients with catatonia at an early stage and actively work towards prevention and focused care, thereby improving patient outcomes to the greatest extent possible. Emergency department nurses can provide targeted health education for high-risk individuals to prevent the occurrence of pneumonia. When receiving such patients with catatonia, nursing vigilance can be heightened to reduce the incidence of pneumonia. Additionally, attention should be given to avoiding re-exposure to risk factors during the pneumonia recovery process. We hypothesize that patients with catatonia are more likely to have bacterial pneumonia. Therefore, this study aims to analyze the risk factors for patients with catatonia in developing pneumonia, and provide feasible preventive nursing recommendations for this patient population.

## 2 Method

### 2.1 Participants

This study is a cross-sectional study. This study included patients admitted to our emergency department for observation from January 2019 to October 2021. A total of 88 patients who met the DSM-5 diagnostic criteria for catatonia were included, with 17 patients in the bacterial pneumonia group and 71 patients in the non-pneumonia group. The pneumonia group was the observation group, while the non-pneumonia group served as the control group. The diagnostic criteria for pulmonary infection (15) included: thick and difficult-to-expectorate sputum, accompanied by fever and cough, with crackles and wet rales heard in both lungs; peripheral blood white blood cell count ≥10×10^9/L or ≤4×10^9/L; chest CT showing evidence of pulmonary infection or chest X-ray suggesting increased lung markings. This study did not include pneumonia caused by the COVID-19 and other causes.

Inclusion criteria: (1) Meet the diagnostic criteria for catatonia according to the DSM-5; the diagnostic criteria for catatonia in the DSM-5 include the presence of at least 3 out of 12 psychomotor features, including catatonia, freezing, waxy flexibility, mutism, negativism, posturing, stereotypy, mannerism, automatic obedience, grimacing, echolalia, and echopraxia (16). (2) Age between 18 and 60 years, regardless of gender. (3) Meet the diagnostic criteria for bacterial pulmonary infection. Exclusion criteria: (1) Elevated inflammatory factors due to other causes, such as trauma, gastroenteritis, urinary tract infection, cancer, etc. (2) Those who meet the diagnostic criteria for other mental disorders in the DSM-5.

### 2.2 Assessment procedures

Collection and compilation of patients’ clinical data, including age, gender, place of residence, height, weight, body mass index (BMI), smoking and drinking history, bedridden days, and scores on the Activities of Daily Living Scale (ADL). Blood test indicators included white blood cell count, neutrophil count, neutrophil percentage, total protein level, and albumin level, which were measured within 3 days of admission or before the onset of pneumonia.

The Activities of Daily Living Scale (ADL), developed by Lawton and Brody in the United States in 1969, consists of a physical self-maintenance scale and an instrumental activities of daily living scale. It is mainly used to assess the daily living abilities of the subjects. The ADL scale consists of 14 items, including 6 items in the physical self-maintenance scale (toilet use, feeding, dressing, grooming, ambulation, and bathing) and 8 items in the instrumental activities of daily living scale (telephone use, shopping, meal preparation, housekeeping, laundry, use of transportation, medication management, and handling finances). Each item is scored from 1 (normal) to 4 (severely impaired), with a maximum total score of 56. The results are classified as follows: a total score less than 16 indicates complete self-care, a total score of 16-21 indicates functional decline, and a total score of 22 or higher or two or more items with a score of 3 or higher indicate significant functional impairment.

Family support is one of the items in the Suicide Risk Factors Assessment Scale. The scale categorizes risk factors into three levels, and family support is one of the factors in the third level, with a score of 0 indicating good support and a score of 1 indicating poor support (17). The caregivers were asked about the patient’s family relationship (good/bad), and married patients were asked about the couple relationship (good/bad) to determine the patient’s family support status.

The included subjects were all hospitalized patients in the emergency observation ward. The inclusion criteria of the patients were referred to the diagnostic criteria for catatonic disorder in DSM-5, and the diagnosis was verified by 2 attending psychiatrist participating in the study. The questionnaire included: age, sex, place of residence, marriage, years of education, occupation, previous medical history, smoking history, drinking history, length of hospitalization, BMI (kg/m2), total protein (g/L), length of bed, white blood cell number, CRP value, and chest CT/X-ray results. The researchers used questionnaires to assess participants’ socio-demographic and disease-related content. The patient’s test results were retrieved from the hospital medical record system and recorded in the questionnaire. The ADL scale is evaluated by asking patients and their caregivers about their conditions before admission and observing patients’ daily life after admission.

All researchers are trained to reach a consensus on the content of the questionnaire variables. Set up research nurses to verify data, manage data preservation, urge researchers to verify, proofread, supervise and modify questionnaires, and input data.

### 2.3 Statistical analysis

The data were analyzed using SPSS 20.0 statistical software. Quantitative data and independent sample t-tests were used for comparisons between the two groups. Qualitative data were expressed as frequencies and percentages, and chi-square tests were used for comparisons between the two groups. Logistic regression analysis was used to identify influencing factors. Point-biserial correlation test was used for correlation analysis. A p-value of less than 0.05 was considered statistically significant.

## 3 Results

A total of 88 patients with catatonia were divided into the pneumonia group (17 cases) and the non-pneumonia group (71 cases), with a pneumonia prevalence rate of 19.32%. The detail of sociodemographic and clinical data between two group see **Table 1**.

**Table 1:** Comparison of demographic and clinical data between pneumonia group and non-pneumonia group.

	Pneumonia group (*n*=17)	Non-pneumonia group (*n* =71)	χ2/Z	P
Age (years)	46.8 (13.7)	38.6 (15.0)	2.051	0.043
Length of hospital stay (days)	13.1 (14.7)	19.3 (16.7)	-1.402	0.164
BMI (kg/m2)	22.5 (4.0)	23.0 (3.9)	-0.552	0.583
Total protein (g/L)	63.1 (6.1)	62.6 (11.5)	0.185	0.853
Gender (n, %)			3.150	0.076
Male	11 (64.7%)	29 (40.8%)		
Female	6 (35.5%)	42 (59.2%)		
Residence (n, %)			0.695	0.404
Urban	11 (64.7%)	38 (53.5%)		
Rural	6 (35.3%)	33 (46.5%)		
Marital status (n, %)			0.938	0.626
Married	10 (58.8%)	36 (50.7%)		
Education (n, %)			5.795	0.215
High school and below	11 (64.7%)	49 (69.0%)		
Employment status			4.178	0.124
(n, %)	12 (70.6%)	33 (46.5%)		
unemployed			0.418	0.518
Alcohol history (n, %)				
Yes	2 (11.8%)	5 (7.1%)		

χ2 represents the chi-square test, Z represents the Mann-Whitney U test.

Univariate analysis was conducted to analyze the following factors: gender, age, place of residence, marital status, education level, occupation, presence of physical illness, smoking, alcohol consumption, BMI, family support, ADL, bedridden days, albumin level, total protein level, etc. The results showed that there were statistically significant differences between the two groups in terms of age, smoking, presence of physical illness, family support, ADL, albumin level, and bedridden days (P < 0.05). The details see **Table 2**.

**Table 2:** Univariate analysis of risk factors for pneumonia in patients with catatonia Variables.

	Pneumonia group (n=17)	Non-Pneumonia group (n=71)	χ2/Z	P
Age (years)	46.8 (13.7)	38.6 (15.0)	2.051	0.043
ADL score	26.6 (12.2)	20.6 (7.6)	2.544	0.013
Albumin (g/L)	36.9 (4.8)	44.4 (10.7)	-2.773	0.007
Bedridden days (days)	2.5 (2.1)	0.56 (1.2)	4.942	<0.001
Physical illnesses (n, %)	12 (70.6%)	25 (35.5)	7.045	0.008
Smoking (n, %)	6 (35.3%)	8 (11.3%)	5.918	0.015
Family support (n, %)	9 (52.9%)	59 (83.1%)	7.103	0.008

Mean (SD); ADL (Activity of Daily Living Scale) is a measure of daily living ability.

A logistic regression analysis was conducted with the occurrence of pneumonia in catatonia patients as the dependent variable (0 = No, 1 = Yes). The significant variables identified in the univariate analysis were used as independent variables. For age, ADL, albumin, and bedridden days, the original values were used for assigning values. For smoking, presence of physical diseases, and family support, the values were assigned as 0 = No and 1 = Yes.

The logistic regression analysis results showed that smoking, bedridden days, family support, and nutritional status were risk factors for pneumonia in catatonia patients (P < 0.05). Specifically, longer bedridden days and smoking were identified as risk factors, indicating that the longer the bedridden days and the more smoking, the higher the risk of developing pneumonia. On the other hand, family support and albumin levels were identified as protective factors, suggesting that better family support and higher albumin levels were associated with a lower risk of pneumonia. The results of the multivariate analysis of risk factors for pneumonia in catatonia patients are shown in **Table 3**.

**Table 3:** Multivariate analysis of risk factors for pneumonia in patients with catatonia.

	Regression coefficient	Standard error	Waldχ^2^	*P*	OR*	95%CI
Albumin level	-0.255	0.108	5.531	0.019	0.775	0.627-0.958
Bedridden days	0.695	0.219	10.052	0.002	2.004	1.304-3.080
Family support	-1.965	0.869	5.115	0.024	0.140	0.026-0.769
Smoking	2.052	0.872	5.537	0.019	7.781	0.026-0.769

*OR, odds ratio; CI, confidence interval.

## 4 Discussion

Patients with catatonia are prone to develop pneumonia due to prolonged bedridden time, poor nutrition, and weakened immune system. Without effective and timely nursing and treatment interventions, it may endanger the lives of patients. Additionally, the prolonged treatment of pneumonia-related physical illnesses can worsen the condition of patients with catatonia, greatly impacting the assessment and treatment of catatonia symptoms. There is a lack of discussion on risk factors and nursing methods for pneumonia in patients with catatonia in existing domestic and foreign literature. This study found a pneumonia incidence rate of 19.32% in patients with catatonia and identified bedridden days, nutritional status, smoking, and family support as risk factors for pneumonia in these patients. This indicates that patients with these risk factors belong to a high-risk group, and we provide feasible preventive nursing recommendations for such patients to reduce the occurrence of pneumonia.

This study found a positive correlation between age, ADL, white blood cells, percentage of neutrophils, C-reactive protein, and pneumonia. The correlation between white blood cells, percentage, C-reactive protein, and pneumonia was stronger, while albumin showed a negative correlation with pneumonia. This finding is consistent with other studies (18–20). We found that the ADL score in the pneumonia group was higher than that in the non-pneumonia group (P=0.013) in the univariate analysis of risk factors. The logistic regression analysis results, We did not find that ADL was a risk factor for pneumonia in catatonic patients. However, previous analysis suggests that patients with prolonged bedridden time have significantly reduced ADL, and as age increases, the body’s functions decline, increasing the risk of pneumonia (18). In future studies, we can conduct a more in-depth analysis of ADL. White blood cells and C-reactive protein are important indicators of infection, and higher levels of white blood cells and C-reactive protein indicate a higher susceptibility to pneumonia (19). Albumin reflects the patient’s nutritional status, and when nutrition is poor, the body mobilizes proteins and amino acids to convert them into glucose to ensure adequate blood sugar supply during stress or infection. Therefore, a decrease in albumin is a slow process, and if it decreases, it indicates poor immune function and a high likelihood of developing pneumonia (20).

This study found that prolonged bed rest is one of the risk factors for developing pneumonia. Previous research has also shown that prolonged bed rest in elderly patients is associated with increased risk of pneumonia, including factors such as bed rest duration and malnutrition (13), which is consistent with the findings of this study. The analysis suggests that prolonged bed rest leads to limited changes in body position and decreased chest movement, resulting in reduced lung ventilation and increased susceptibility to obstruction of the terminal bronchi by respiratory secretions, as well as inflammation caused by pulmonary edema at the base of the lungs (21). As patients with prolonged bed rest have decreased daily activities and require partial or complete assistance in their daily lives (22), inadequate nursing care can worsen the severity of pneumonia and potentially endanger their lives. Patients with catatonia are similar to patients with prolonged bed rest in that their psychiatric symptoms often result in prolonged bed rest, partial or complete dependence in daily activities, and difficulty in expectorating sputum and secretions, which can lead to the occurrence of pneumonia (21). Additionally, psychiatric patients have weakened immune systems, which also contributes to the accelerated development of pneumonia. Previous studies have found that psychiatric patients are more susceptible to infection due to sleep disorders and behavioral disturbances (23), and long-term use of antipsychotic drugs can cause adverse reactions such as leukopenia, leading to decreased resistance to infection. Therefore, targeted care, including bed rest care, should be provided in a timely manner, with a focus on reducing the duration of bed rest, turning and patting the patient’s back daily, and assisting with sputum clearance. During sputum clearance, patients should be guided to cough actively and use diaphragmatic breathing, and their back can be percussed from bottom to top to assist with sputum clearance. For patients with thick and difficult-to-expectorate sputum, nebulization therapy with ambroxol plus saline solution can be administered, which has a significant expectorant effect. Nebulization therapy should be conducted 3-4 times a day, with a dosage of 5-10mL each time. Additionally, to reduce the risk of food or secretion reflux, patients can be positioned alternately between lateral and supine positions, with the head of the bed elevated at a 30° angle during supine position, and liquid or semi-liquid diet can be consumed in this position to prevent choking during swallowing and reduce the occurrence of pneumonia.

The study found that respiratory tract infections are the most common nosocomial infections among psychiatric patients (24). 70% of pneumonia-causing pathogens originate from the oral-pharyngeal region, and the retention of oral secretions allows colonized bacteria to enter the lungs and trigger pneumonia (25). Patients with catatonia are at an increased risk of respiratory tract infections due to increased oral secretions or suppression of respiratory ciliary movement caused by the use of antipsychotic drugs. Bedridden patients have limited ability to brush and rinse their teeth, leading to rapid growth and proliferation of bacteria in the oral cavity, increasing the risk of bacterial pneumonia infection (26). Oral care can remove phlegm crusts and residual debris in difficult-to-reach areas adhered to teeth and gums, reducing the number of remaining dental plaque (25), thus reducing the occurrence of pneumonia. Research has shown that intensive oral care has a significant preventive effect on aspiration pneumonia in critically ill comatose patients, as effective oral care reduces the bacterial content in the oral cavity, thereby reducing the incidence of pneumonia (27). The method of oral care involves daily removal of pharyngeal secretions and wiping the oral cavity twice a day with compound chlorhexidine to reduce oral bacterial growth.

In this study nutritional status is one of the risk factors for developing pneumonia. Patients with catatonia may refuse food and water under the domination of psychiatric symptoms, resulting in poor physical nutrition, which is also one of the causes of pneumonia. Albumin, hemoglobin, and potassium are indicators for assessing nutritional status, and low levels of these indicators indicate malnutrition. Research has shown that during periods of poor nutrition, the body mobilizes proteins and amino acids to convert into glucose to ensure blood glucose supply during stress or infection. Therefore, a decrease in albumin levels indicates malnutrition and a decrease in immune function, which increases the risk of pulmonary infection in elderly bedridden patients (20). The above research findings are consistent with the results of this study. The analysis suggests that patients with prolonged bed rest have reduced gastrointestinal motility, leading to reduced nutrient absorption. The decreased energy intake due to reduced supplementation and increased disease expenditure results in decreased protein synthesis and increased protein breakdown, leading to a decrease in protein levels in the body, which in turn leads to malnutrition, decreased immune function, and the occurrence of pneumonia (28). Therefore, strengthening nutritional support, ensuring adequate total energy intake, implementing effective protective isolation, and reducing the risk of pneumonia are necessary.

Meanwhile, our study found that smoking is one of the risk factors for developing pneumonia. Research has found that smoking is an independent risk factor for lung infections in elderly bedridden patients and is a-known important risk factor for the development of various diseases among various personal lifestyles (29). This is consistent with the findings of this study. The toxic factors produced by smoking can damage lung tissue cells and even induce cell apoptosis. Long-term smoking can also impair the defense barrier function of cells, leading to inflammation and stimulating the proliferation of submucosal glands, obstructing the drainage of respiratory secretions and bacteria, and causing lung infections (30). In the context of research advances on the relationship between smoking and COVID-19, it has been proposed that smoking can weaken the mucociliary clearance ability, increase mucus secretion, impair endocytosis in pulmonary epithelial cells, and decrease immune function (31, 32). Therefore, reducing or quitting smoking, improving immune function, and reducing lung damage are important in reducing the risk of pneumonia.

Interestingly, family support is one of the risk factors for developing pneumonia in our study. Patients with catatonia are prone to pneumonia, and if not detected and effectively cared for before admission, patients may be referred to the respiratory department for treatment of pneumonia, delaying psychiatric diagnosis and treatment and exacerbating psychiatric symptoms. Therefore, family support and care are essential. Previous studies have shown that family members’ attention to changes in patients’ physical conditions and their psychological care are key factors in reducing the incidence of pneumonia and providing nursing care, which is consistent with the results of this study (33). Family support mainly manifests in psychological care and routine care for early prevention of pneumonia. Patients with psychiatric disorders often exhibit suspicion, irritability, defiance, and impulsive behavior, making psychological care even more important. First, provide patients with a comfortable and quiet environment. Secondly, be patient and tolerant in nursing care to gain the patient’s trust. Finally, family members need to actively communicate with the patient, inform them of the relevant factors and reasons for the occurrence of pneumonia, and alleviate the patient’s anxiety. Routine care for pneumonia prevention by the family primarily involves monitoring the patient’s health status and providing timely assistance with turning and patting the patient’s back.

Unfortunately, our study also has some limitations. Firstly, this study is a cross-sectional survey, which may have some recall bias, and the comparison was only made in the population of people with mental disorders. And the causal relationship of its results is still unclear, thus requiring validation by more prospective research. Secondly, there were limitations in the inclusion of risk factors, risk for treatment and prognosis of pneumonia was not discussed, and the assessment of family support was not quantified. Thirdly, due to the limitations of clinical trials in psychiatric hospitals, no test results for biomarkers other than CRP were provided to further clarify the disease classification that causes catatonia. Finally, the sample size is small, and all samples are from a tertiary psychiatric hospital, which may not represent psychiatric patients from different regions and ethnic groups. Despite the limitations, this study provides valuable insights into the care of patients with catatonia combined with pneumonia in psychiatric emergency departments, and offers practical preventive care measures, which can serve as a reference for emergency nursing care.

Despite its limitations, this study is still one of the few studies focusing on nursing care for patients with catatonia in the psychiatric emergency department who develop pneumonia. It provides targeted preventive nursing measures and contributes to the reference value for emergency nursing.

## Data Availability

The raw data supporting the conclusions of this article will be made available by the authors, without undue reservation.
